# Function-informed transcriptome analysis of *Drosophila *renal tubule

**DOI:** 10.1186/gb-2004-5-9-r69

**Published:** 2004-08-26

**Authors:** Jing Wang, Laura Kean, Jingli Yang, Adrian K Allan, Shireen A Davies, Pawel Herzyk, Julian AT Dow

**Affiliations:** 1 Division of Molecular Genetics, Institute of Biomedical and Life Sciences, University of Glasgow, Glasgow G11 6NU, UK; 2 Sir Henry Wellcome Functional Genomics Facility, University of Glasgow, Glasgow G12 8QQ, UK

## Abstract

**Background:**

Comprehensive, tissue-specific, microarray analysis is a potent tool for the identification of tightly defined expression patterns that might be missed in whole-organism scans. We applied such an analysis to *Drosophila melanogaster *Malpighian (renal) tubule, a defined differentiated tissue.

**Results:**

The transcriptome of the *D. melanogaster *Malpighian tubule is highly reproducible and significantly different from that obtained from whole-organism arrays. More than 200 genes are more than 10-fold enriched and over 1,000 are significantly enriched. Of the top 200 genes, only 18 have previously been named, and only 45% have even estimates of function. In addition, 30 transcription factors, not previously implicated in tubule development, are shown to be enriched in adult tubule, and their expression patterns respect precisely the domains and cell types previously identified by enhancer trapping. Of *Drosophila *genes with close human disease homologs, 50 are enriched threefold or more, and eight enriched 10-fold or more, in tubule. Intriguingly, several of these diseases have human renal phenotypes, implying close conservation of renal function across 400 million years of divergent evolution.

**Conclusions:**

From those genes that are identifiable, a radically new view of the function of the tubule, emphasizing solute transport rather than fluid secretion, can be obtained. The results illustrate the phenotype gap: historically, the effort expended on a model organism has tended to concentrate on a relatively small set of processes, rather than on the spread of genes in the genome.

## Abstract 2

Analysis of the transcriptome of the *Drosophila melanogaster* Malpighian (renal) tubule gives a radically new view of the function of the tubule, emphasising solute transport rather than fluid secretion.

## Background

Microarrays allow the interrogation of the transcriptome, the set of genes transcribed in a particular cell type under a particular condition [1]. Arrays are particularly potent tools when their coverage is relatively comprehensive, based on a completed and well annotated genome, such as that of *Drosophila *[2]. Commonly, they are used in time series, for example of development, of life events such as metamorphosis [3], of rhythmic behavior [4] or of responses to environment, such as aging or starvation [5,6]. In *Drosophila*, arrays are frequently used for whole-organism studies, but in multicellular organisms the ease of experimentation must be balanced against two potential problems: sensitivity and opposing changes. In the first case, even large changes in gene expression in a small tissue will not significantly influence the overall levels in the whole organism; in the second, changes in opposite directions in roughly balanced populations of cells (for example, the sharpening of expression patterns of pair-rule genes) will cancel out at an organismal scale. It is thus vital to resolve gene expression not only over time but also over space. In practice, this means looking at gene expression in defined cell types and tissues as well as in the whole organism. Our assumption is that the expression of many putative genes will go undetected until such tissue-specific studies are performed [7] - with obvious consequences for post-genomics - and we illustrate this point in this paper.

We applied Affymetrix arrays in the context of a defined tissue with extensive physiological characterization, the Malpighian (renal) tubule of *Drosophila melanogaster*. The tubule is a valuable model for studies of both epithelial development and function. Developmentally, the tissue is derived from two distinct origins: an ectodermal outpushing of the hindgut and subsequent invasion (late in embryogenesis) by mesodermal cells [8]. Tubule morphology is very precisely and reproducibly specified; in the tiny tissue of 150 cells, there are altogether six cell types and six regions, specified to single-cell precision [9]. The transport processes that underlie fluid production in the tubule are known in extraordinary detail for so small an organism [10-12]. The dual origin of the cell types is reflected by dual roles for the ectodermal principal cells and mesodermal stellate cells in the mature tubule; the principal cell is specialized for active transport of cations, whereas the stellate cell appears to control passive shunt conductance [11,13,14]. Cell signaling pathways are also understood in considerable detail: several peptide hormones that act on tubule have been identified [15-17], and the second messengers cyclic AMP, cyclic GMP, calcium and nitric oxide have all been shown to have distinct roles in each tubule cell type [10,18-20].

This wealth of physiological knowledge provides a framework for the analysis of the results, and thus - unusually in genetic model organisms - a reality check on the usefulness of the experiment.

## Results

The principle of the experiment was to compare the transcriptome of 7-day adult *Drosophila melanogaster *Malpighian (renal) tubules, for which defined state there is a wealth of physiological data, with matched whole flies. As described in Materials and methods, data were analyzed by Affymetrix MAS 5.0 software, or by dChip, or dChip and Significance Analysis of Microarrays (SAM) software. Both methods of identifying differentially expressed genes from dChip-normalized data gave virtually the same results. Indeed, SAM analysis followed by further filtering produced 1,465 differentially expressed genes compared to 1,455 genes identified within filtering by dChip alone. Furthermore, the latter list is indeed a subset of the former one. For that reason we report only the list generated by dChip in comparison with MAS data.

Both MAS and dChip/SAM gave comparable views of the data, despite the radically different approaches to analysis. It has been shown that the average absolute log ratios between replicate arrays calculated with dChip are significantly lower than one calculated with Affymetrix software (Li and Wong [21]). This bias affecting fold-change calculations is the price of the increased precision that manifests itself in reduced variance, and consequently in the increased sensitivity of identification of differentially expressed genes. Nonetheless, the rank correlation is good (Spearman's *r *= 0.6, *p *< 0.0001). Taking genes called as significant by both systems, MAS5 'up' call or dChip *t*-test *p*-value of 0.01, and narrowing the list by setting an arbitrary cutoff of twofold enrichment and minimum mean difference of 100, MAS5 reported 683 genes and dChip reported 671. Furthermore, the dChip-reported genes overlap with 77% of MAS5-reported genes and this number increases to 91% if only the top 500 MAS5-reported genes are considered. Our confidence in the quality of the dataset is thus high. For simplicity, and because the two analyses produce concordant results, further analysis is restricted to the MAS5 results.

**Figure 1:**
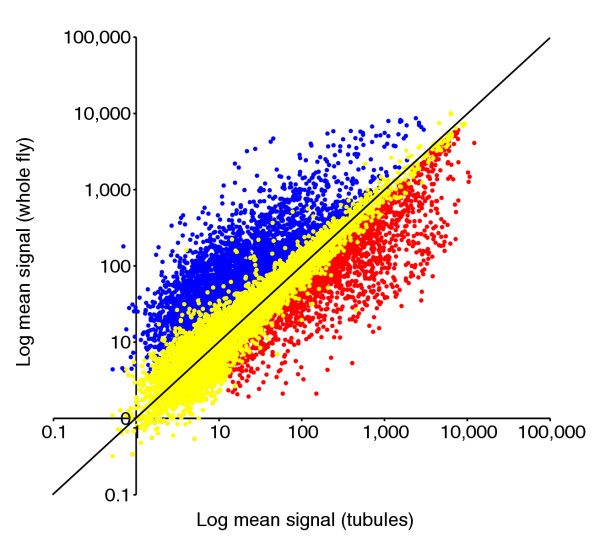
Scatterplot of mean whole fly vs tubule signal intensities. Genes called as significantly enriched in tubule compared with fly by MAS 5.0 are in red, those significantly depleted in blue, and those not significantly different in yellow.

The full microarray data have been deposited in ArrayExpress [22]. The fly versus fly and tubule versus tubule samples were extremely consistent, despite the technical difficulty in obtaining the latter (30,000 tubules were dissected in total). In contrast, there was wide divergence between fly and tubule samples (Figure 1). Although a common set of housekeeping genes showed comparable abundance, there was a large set of genes enriched in the fly sample, and a smaller set of genes strongly enriched in the tubule sample. In detail, of 13,966 array entries, 6,613 genes were called 'present' in all five fly samples, compared with 3,873 in tubules. A total of 3,566 genes were present in both fly and tubule: 3,047 in fly only and 307 in tubule only. This illustrates the point that whole-organism views of gene expression are not necessarily helpful in reflecting gene-expression levels in individual tissues. The microarray data are summarized in Tables 1,2.

### Validation of the microarray

Four genes were selected from each of three fly tubule expression classes: very highly enriched; uniformly expressed; and very highly depleted. The expression of each gene was verified by quantitative reverse transcription PCR (RT-PCR) and the data are presented in Table 3. The agreement between Affymetrix microarray and quantitative PCR determination is good, further increasing our confidence in the robustness of the dataset, and in the approximate correspondence between signal and RNA abundance as a population average. It should be noted that the absolute sizes of the ratios are quite variable; this is a property of dividing a large number by a very small one. Nonetheless, genes scored as enriched or depleted on the arrays are invariably similarly scored by quantitative RT-PCR (QRT-PCR).

**Figure 2:**
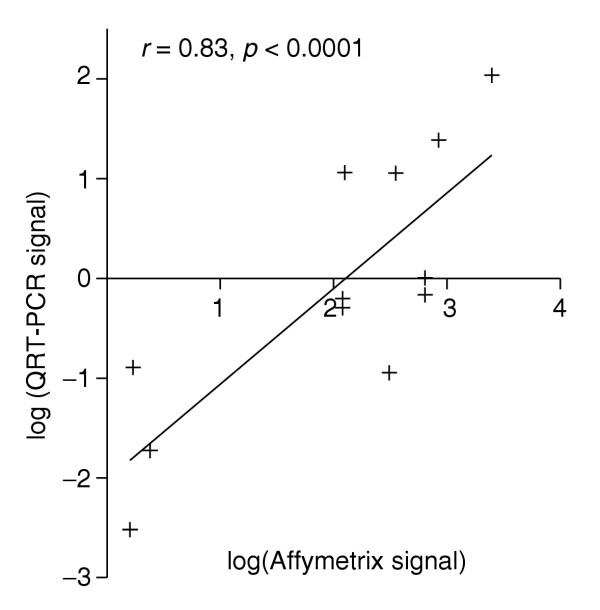
Semi-quantitative inter-gene comparison is possible using Affymetrix signal. The 24 QRT-PCR results underlying Table 3 were normalized against *rp49*, and plotted against the Affymetrix signal globally normalized as in MAS 5.0. Spearman's *r *was calculated, and significance of the correlation assessed (one-tailed), using Graphpad Prism 3.0.

These data can also be used to validate the use of the normalized Affymetrix signal as a semi-quantitative measure of RNA abundance (Table 1). If the QRT-PCR dataset of Table 3 is normalized against corresponding signals for *rp49 *(generally taken to be a ubiquitous gene with invariant expression levels in *Drosophila*), and compared with the globally normalized Affymetrix signal, the agreement is seen to be excellent (Figure 2), with a Spearman's *r *of 0.83 (*p *< 0.0001). With appropriate caution, the normalized Affymetrix signal can thus be taken as a reasonable estimate of expression levels between genes.

Table 1 shows the top 20 genes listed by mean Affymetrix signal intensity. Although this is only a semi-quantitative measure of transcript abundance, the identities of the known genes in the lists are illuminating, and persuade us that the approach has some informal value. Specifically, mRNAs for ribosomal proteins dominate the list, and transporters are conspicuous in the balance. For example, the V-ATPase that energizes transport by tubules is represented by one gene (other subunits are also abundant, but just below the cutoff for Table 1). The α-subunit of the Na^+^, K^+ ^ATPase is also highly abundant: this is more surprising, and is discussed below. Two organic cation transporters are also very abundant. Alcohol dehydrogenase, long known to be expressed in tubules [23,24], is also a major transcript. There are also surprises: the most abundant signal is for metallothionein A. This is entirely consistent with our classical understanding of tubule function: it has long been known as a route for metal sequestration and excretion [25-30]. However, in the entire literature on Malpighian tubules, we are not aware of a physiological investigation of the role of metallothionein, other than documentation of expression [31,32]. The microarray results can thus potently direct and inform future research.

Table 2 lists the 53 tubule-enriched genes that are enriched at least 25-fold, in comparison with the whole fly (the full list is provided as an additional data file). The conspicuous feature of these data is the extent to which tubule transcripts differ from any previously published profile. When comparing fly with tubule, there is a large set of genes that are downregulated and another large set of genes that are upregulated in tubule. The extent of the upregulation is also remarkable: the top gene is 99-fold enriched; the top 10 at least 50-fold enriched; and the top 100 at least 16-fold enriched in tubule compared to fly. The standard errors are also extremely low, meaning that we can be very confident (by two separate statistical measures) of the genes called significantly enriched in tubule.

### The phenotype gap

**Figure 3:**
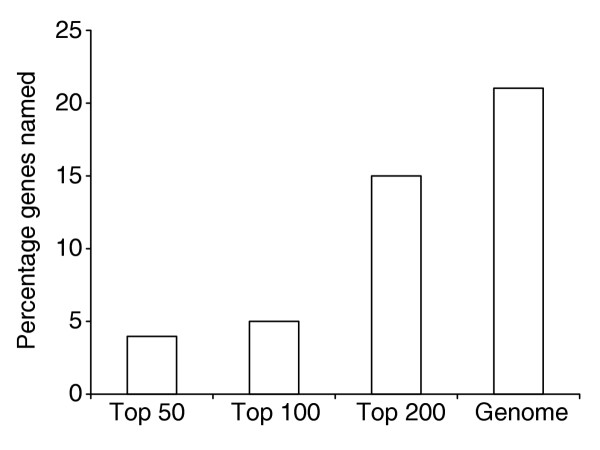
The phenotype gap. Genes enriched in tubules are historically under-researched. The percentage of genes with explicit names (other than automatic CG annotations) is shown for the entire genome, and for the top 50, 100 and 200 genes (as judged by fold enrichment) from the tubule dataset.

Another prominent feature of the signal data in Table 1 is the relatively large fraction of novel genes (those for which there is not even a computer prediction of function) at the top of the list. Indeed, five of the top 10 genes by signal intensity are completely novel - that is, there are no known orthologs - and should provide tantalizing insights into tubule function. The 'phenotype gap' [33,34] is a key problem in functional genomics; that is, the genetic models preferred for genomics are historically not the organisms selected by physiologists. This can lead to a log-jam in reverse genetics, which depends critically on a wide range of phenotypes to identify effects of the mutation of target genes [12]. It has recently become possible to quantify the phenotype gap [35]. The present dataset elegantly exposes the phenotype gap in *Drosophila*, and shows that the tubule phenotype may go some way to closing it. Around 20% of *Drosophila *genes have been studied in sufficient detail to attract names (beyond the standard 'CG' notation for computer-annotated genes). Figure 3 shows that the fraction of anonymous genes in the tubule-enriched list is far higher than would be expected. That is, previous work has tended to overlook these genes. Conversely, because it is possible to perform detailed physiological analysis in tubules, it is possible to close the phenotype gap for these genes. There is a general implication from these data: that functional genomics, in *Drosophila *and other species, will rely increasingly on the study of specific tissues, as it is only in this context that expression of genes will be either measurable or explicable.

### Reconciling array data with function

Many microarray experiments merely classify enriched genes to their Gene Ontology families. However, the uniquely detailed physiological data available on the Malpighian tubule allows a much more informative approach. The dataset can be validated by inspection, based on known molecular functions in the tissue and new functions can be inferred from abundant or enriched transcripts in the dataset. As the array is relatively comprehensive (corresponding to the 13,500 genes in release 1 of the Gadfly annotation), the results are also relatively authoritative.

#### Organic solutes

The housekeeping ribosomal transcripts vanish from the enrichment list (Table 2), which is now dominated by transporters. Intriguingly, these are not for the V-ATPase that is considered to dominate active transport by the tubule, but for organic and inorganic solutes. There is a range of broad-specificity transporters - for organic cations, anions, monocarboxylic acids, amino acids and multivitamins. There are also multiple inorganic anion co-transporters for phosphate and iodide. Most are not only very highly enriched, but also highly abundant. In more detail, the results are remarkable (Table 4). Nearly every class of transporter is represented, and almost all of these have at least one representative that is both abundant and enriched, implying a very specific renal role; indeed, this table contains the genes with the highest average enrichments of any class, frequently more than 30-fold. Some transporters have been documented implicitly as having a tubule role; many of the classical *Drosophila *eye-color mutants also have an effect on tubule color, and have since been shown to encode genes for transport of eye-pigment precursors [12,36]. These genes now turn out to be both abundant and enriched; among the ABC transporters are *scarlet *and *white*, and among the monocarboxylic acid transporters is *CG12286*, which we have recently argued to correspond to karmoisin, a probable kynurenine tranporter [37]. Glucose and other sugar transporters are consistently abundant and enriched, implying that sugar transport is a major (and previously unsuspected) role of the tubule. Inorganic transporters are also included in the table; there are also copper and zinc transporters, which is consistent with electron-probe X-ray microanalysis data that heavy metals accumulate in tubule concretions [38,39], and with the extreme abundance of metallothionein A (Table 1).

As well as specific transporters, the tubule is enriched for several families of broad-specificity transporters (organic anion and cation transporters, multivitamin transporters, ABC multidrug transporters and an oligopeptide transporter). When combined these would be capable of excreting a huge majority of organic solutes. These results invite a substantial revision of our interpretation of the role of the tubule. Classically, it is considered to be the tissue that excretes waste material, both metabolites and xenobiotics, and provides the first stage of osmoregulation. However, nearly all work on insect tubules in the last half-century has focused on the ionic basis of fluid secretion and its control, as these are easily measured experimentally. Although there have been sporadic reports on the active transport of organic solutes such as dyes [40-42], the historical view was of a relatively leaky epithelium, with a paracellular default pathway for those solutes not recognized by specific transporters. While consistent with the more classical view of the tubule, our results also suggest that the insect is emulating a leaky epithelium to produce the primary urine by incorporating a vast array of broad-specificity active transporters in the plasma membranes of what is electrically rather a tight epithelium. Indeed, this interpretation is consistent with other independent data: the intercellular junctions in tubule are known to be of the pleated stellate variety, the invertebrate equivalent of tight junctions [43]; and, like salivary glands, tubule cells are known to be highly polytene [44-47] or even binucleate [48], adaptations that maximize the size of cells and thus maximize their area/circumference ratios.

#### V-ATPases

Physiological analysis of the tubule has concentrated on the secretion of primary urine, and the energizing transporter is a plasma membrane proton pump, the V-ATPase [13,49-51]. This is a large holoenzyme of at least 13 subunits, encoded by 31 *Drosophila *genes [52,53]. V-ATPases have two distinct roles, one carried out at low levels in endomembrane compartments of all eukaryotic cells and the other in the plasma membranes of specialized epithelial cells of both insects and vertebrates [54]. In such cells, the V-ATPases can pack the plasma membrane to such an extent that they resemble semi-crystalline arrays when observed by electron microscopy [55]. It is clearly of interest to find out which genes contribute to the plasma-membrane role of the V-ATPase, though this would normally involve difficult and tedious generation of selective antibodies capable of distinguishing between very similar proteins. However, the mRNAs for those V-ATPase subunits enriched in epithelia should also be particularly abundant; one could thus predict that at least one gene encoding each V-ATPase subunit should show enrichment in tubule compared with the rest of the fly. This is indeed the case (Table 5): invariably, one gene for each subunit is both significantly enriched, and far more abundant, than any other gene encoding that subunit. The reason that the enrichment is not higher is probably because the whole-fly samples contain other epithelia, each with enriched V-ATPase, as minor parts of the overall sample.

The array data thus allow a rapid and authoritative prediction to be made on the subunit composition of the plasma membrane V-ATPase. It will be interesting to extend these data to other epithelia in which V-ATPase is known to be functionally significant.

#### Na^+^, K^+^- ATPase

The role of the classical Na^+^, K^+^-ATPase in tubule is enigmatic. In nearly all animal epithelia, transport is energized by a basolateral Na^+^, K^+^-ATPase, which establishes a sodium gradient that drives secondary transport processes. By contrast, insect epithelia are energized by a proton gradient from the apical V-ATPase [56,57] and, consistent with this, many insect tissues are paradoxically refractory to ouabain, the specific Na^+^, K^+^-ATPase inhibitor [58]. Accordingly, models of insect epithelial function tend not to include the Na^+^, K^+^-ATPase. It is thus interesting to note that both *Atpalpha *and *Nervana 1 *(encoding isoforms of the α and β subunits, respectively) are among the most abundant transcripts in tubule (Table 6). Both are about as enriched in tubule as the V-ATPase subunits, but are significantly more abundant (compare Table 5). By contrast, a novel alpha-like subunit (*CG3701*), and both *Nrv2 *(the neuronal β-subunit) and other novel β-like subunits are at near-zero levels. As Na^+^, K^+^-ATPase has previously been documented as being particularly abundant in *Drosophila *tubule [59], it may thus be prudent to re-include the Na^+^, K^+^-ATPase as an important part of models of tubule function.

#### Potassium channels

Potassium is actively pumped across the tubule, and the main basolateral entry step is via barium-sensitive potassium channels, both in tubule [50,60,61] and in other V-ATPase-driven insect epithelia [62,63]. Of the ion channels, the potassium channel family is by far the most diverse in all animals: in *Drosophila*, there are at least 28, and in human 255, K^+^-channel genes [64]. Inspection of the potassium channels on the array (Table 7) clearly identifies just four that are expressed at appreciable levels. *Irk3*, *Ir*, *Irk2 *and *NCKQ *are all both very abundant and highly enriched in tubule. *Irk3 *in particular is 80-fold enriched over the rest of the fly, implying a unique role in tubule. Three of these genes are members of the inward rectifier family of potassium channels: supporting the hypothesis that they are critical for potassium entry, these channels are known to be highly barium-sensitive [65]. An inward rectification of potassium current (meaning that potassium would pass much more easily into the cell than out) would be ideal for a basolateral entry step. Inward rectifier channels normally associate with the sulfonylurea receptor (SUR), an ABC transporter, in order to make functional channels [66,67]. In tubules, *SUR *mRNA is present at extremely low abundance (signal 6, enrichment 0.9 times). However, *CG9270*, a gene with very close similarity to *SUR *(1 × 10^-28 ^by BLASTP) is very abundant in tubule (see Table 4), (signal 422, enrichment 21 times). A second very similar gene, *CG31793 *(previously also known as *CG10441 *and *CG17338*), is very much less abundant (signal 24, enrichment 0.5). We therefore predict that novel inward rectifiers, formed between *Irk3*, *Ir *or *Ir2 *and *CG9270*, may provide the major basolateral K^+ ^entry path in tubule. In contrast, the other classes of K^+ ^channel, and the Na/K/Cl co-transporter that has been documented in tubule, are all relatively low in both abundance and enrichment.

#### Chloride and water flux

In a fluid-secreting epithelium, a necessary correlate of the active transport of cations must be the provision of a shunt pathway for anions and a relatively high permeability to water. In *Drosophila *tubules, a hormonally regulated chloride conductance pathway has been shown to occur in the stellate cells, although the molecular correlate of the currents has not been determined. There are three ClC-type chloride channels in the *Drosophila *genome, and RT-PCR has shown that all three are expressed in tubule [12]. The array data present a prime candidate (Table 8). Although all three genes are expressed, only one (*CG6942*) is both very abundant and enriched in tubule (signal 251, enrichment 4). It is thus an obvious candidate partner to provide a shunt pathway for the epithelial V-ATPase.

Water flux through the tubule is also phenomenally fast: each cell can clear its own volume of fluid every 10 seconds [12]. Although traditionally it was thought that only a leaky epithelium could sustain such rates, the identification of aquaporins (AQP) (the predominant members of the major intrinsic protein (MIP) family) as major water channels in both animals and plants [68] provides an obvious counter-explanation. There is physiological and molecular data for the presence of aquaporins in *Drosophila *tubule [69], and AQP-like immunoreactivity has been demonstrated in stellate cells [12]. Table 9 shows that only four of the seven AQP/MIP genes are abundant, and only three enriched. One can thus tentatively assign an organism-wide role to *CG7777 *(signal 243, enrichment 0.6), but tubule-specific roles to *CG4019*, *CG17664 *and *DRIP*. In particular, *CG17664*, is both highly abundant and very highly enriched (signal 705, enrichment 7.9).

### Control of the tubule

The hormonal control of fluid secretion is well understood. The major urine-producinig region of the tubule is the main segment [70], and is composed of two major cell types, principal and stellate cells [9,13,71]. Active cation transport in the principal cell is stimulated by the hormones calcitonin-like peptide and corticotrophin releasing factor (CRF)-like peptide, both of which act through cyclic AMP (cAMP). Another peptide family, the CAPA peptides, act through intracellular calcium to stimulate nitric oxide synthase and thus raise cyclic GMP (cGMP), an unusual autocrine role for nitric oxide [20,72]. In the stellate cell, the chloride shunt conductance is activated by leucokinin [17,73], and a role for tyramine as an extracellular signal has also been proposed [74]. So far, the CAPA and leucokinin receptors have been identified [75,76]; both are prominent among the receptors enriched in tubule (Table 10). The CAPA receptor appears much more highly enriched in tubule than the leucokinin receptor, which is consistent with our understanding of each: the tubule is the only known target of CAPA, whereas leucokinin receptors are widely distributed in the adult gut, gonad and nervous system [75].

There are many other receptors that are reasonably abundant and enriched in tubule. As well as candidate receptors for calcitonin-like and other neuropeptides, there are two glycine/GABA-like receptors that might be expected to form ligand-gated chloride channels, together with good matches to vascular endothelial growth factor-like, insulin-like and bombesin-like receptors. The localization of, ligands for, and functional roles of these receptors will be of great interest. It should be noted in this context that all hormones characterized so far act on one of the two main cell types in the principal section of the tubule. There are, however, six genetically defined cell types and six regions in the adult tubule [9], and it is likely that there will at least be ligands acting on the initial segment to stimulate calcium excretion, and others acting to regulate reabsorption by the lower tubule. If any of these receptors maps to these regions, they would be prime candidates for such roles.

Overall, the main surprise from these data is the sheer range of candidate ligands that could be inferred; this more than doubles the size of the endocrine repertoire so far postulated for insect tubules.

On a more general level, it is possible to trace out the key genes in all three intracellular signaling pathways that have been studied in detail in *Drosophila *tubule (Table 11). The results for signaling genes tend not to be as clear-cut as for transporters, as many are rather widely distributed, and so do not show enrichment, and many do not require high standing levels of protein (and implicitly mRNA) to achieve their effects. Nonetheless, it is possible to identify genes that are at least present, and frequently enriched, in tubule. For the cAMP pathway, it is possible to identify adenylate cyclases, protein kinase A catalytic and regulatory subunits, and a phosphodiesterase (*dunce*). For cGMP, there are both soluble and membrane guanylate cyclases, implying that the tubules may produce cGMP directly in response to novel ligands, as has recently been suggested [77]. Both *Drosophila *genes encoding protein kinase G are expressed in tubule, and one is highly enriched. This is consistent with the renal phenotype observed both in *foraging *mutants [78], and in tubules in which protein kinase G is overexpressed [79]. There is also a PDE11-like phosphodiesterase. For calcium, two genes for phospholipase C, one for calmodulin, and one for protein kinase C and for calcium/calmodulin-dependent protein kinase are apparent. There are also a number of interesting modulatory or anchoring proteins, such as 14-3-3 zeta, A-kinase anchoring proteins, and receptors for activated C-kinase (*Rack1*).

### How is the tubule specified?

The developmental origin of the tubule has been reviewed in detail [80-82]. Briefly, four unique 'tip cells', specified by a cascade of neurogenic genes, control cell division in four outpushings (anlagen) of the hindgut, to form the Malpighian tubules. Late in embryogenesis the tubule is invaded by mesodermal cells, which intercalate between the future principal cells, and which then differentiate to form stellate cells [8]. In the adult, there are known to be at least six cell types and six tubule regions [9]. These regions are specified to great precision, and it is clear that each cell in the tubule has a precise positional identity. How does this identity persist throughout the lifetime of the animal? Presumably, combinations of transcription factors interact to provide both regional and cell-type coordinates and, after early establishment, these combinations must persist into adulthood. The microarray data allow the identification of transcription factors that are either highly abundant or highly enriched in tubule. Although this is by no means a complete list of transcription factors that are of importance to tubules, it is a good starting point. Furthermore, there are enhancer trap or reporter gene constructs available for many transcription factors. Accordingly, the top transcription factors and DNA-binding proteins were identified from the array dataset (Table 12).

Some of these transcription factors are already known to be present in tubule, and their presence is confirmed: *cut*, which is known to be required for development of, and expressed in adult Malpighian tubules [83]; and *forkhead *and *homothorax*, both implicated by expression or mutational analysis to be involved in tubule development [84,85]. *Teashirt*, which has recently been shown to be stellate-cell specific in the late embryo [8], is also present in the adult, with fairly high enrichment (4.6 times).

The array results also implicate a further set of transcription factor genes (*ETS21C*, *CG4548, bowl*, *sequoia*, *tap*, *CG1162*, *pnt*, *shaven*, *forkhead domain 59A*, *sloppy paired 2*, *lim3*) as important in adult. Significantly, these mainly encode transcription factors implicated in development of the nervous system (another ectodermal tissue), so their reuse in the adult tubule is not too surprising. Once the binding sites for these factors are known, it will be interesting to model gene expression in different tubule regions.

**Figure 4:**
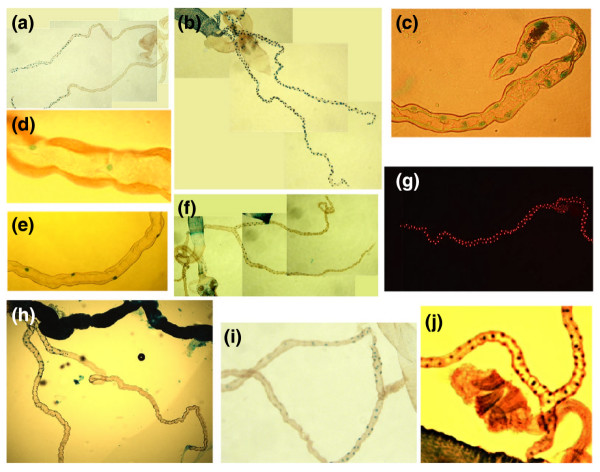
Expression patterns in tubules of some of the transcription factor genes indicated by the microarray data as being expressed in tubules. **(a) ***homothorax *(*hth*^05745^), principal and stellate cells of initial and transitional segments only; **(b) ***polyhomeotic proximal *(*ph-p*), all cells of tubule, and midgut; **(c) ***pointed *(*pnt*^1277^), principal and stellate cells of initial and transitional segments only; **(d) ***corto *(*corto*^07128*b*^), stellate cells only; **(e) ***teashirt *(*tsh*^04319^, a kind gift of H. Skaer), stellate cells only; **(f) ***bunched *(*bnc*^00255^), principal cells, whole tubule; **(g) ***cut *(immunocytochemistry, antibody a kind gift of Jan lab), whole tubule, principal cells only; **(h) ***CG7417 *(*CG7417*^201*Y*^), lower tubule (and midgut - strong); **(i) ***arc *(*a*^*k*11011*b*^), lower tubule, not ureter; **(j) ***Stat92E*^06346^, all tubule cells and midgut.

As transcription factors have been studied experimentally in some detail, they are relatively well represented by enhancer trap and other *in vivo *construct lines. Although individual lines do not necessarily represent the complete expression pattern of their cognate genes, a collection of such lines can provide a rapid first validation of a gene list (Table 12). Accordingly, representative reporter gene lines were ordered from the Bloomington Stock Center [86], and their adult staining patterns in tubule and gut are shown in Figure 4. The results are exciting: most lines showed patterned staining in tubule that is consistent with our original genetically derived map of the tubule [9]. For example, *homothorax *marks out the initial, main and transitional segments of the tubule, whereas *CG7417 *marks the complementary lower tubule domain. The latter line is widely used as a highly specific mushroom body *GAL4 *driver line in brain, and it is interesting that the two known lower tubule *GAL4 *driver lines (c507 and c232) are both insertions in *alkaline phosphatase 4*, a gene which is only expressed in lower tubule and the ellipsoid bodies of brain (next to the mushroom bodies) [87]. There is also a cell-type-specific transcription factor: *corto *is found only in stellate cells. Several other transcription factors show ubiquitous, rather than patterned, expression in the tubule, but this is nonetheless consistent with their identification in the microarray dataset.

Another interesting aspect of the data in Table 12 is the number of anonymous CG genes implicated in tubule function. These genes have been annotated as transcription factors because of DNA-binding domains, for example, but have not been characterized functionally. The epithelial phenotype gap is thus evident even in this most intensely studied group of genes.

### Exceptions to the rule

The whole premise of microarray work is that an abundant or enriched signal indicates the importance of a gene product in a particular context. This hypothesis is normally both untested and unchallenged. The unusual depth of functional understanding of the tubule allows a more rigorous appraisal. In fact, the majority of the genes implicated in tubule function are found well up the list. There are, however, several conspicuous exceptions (Table 13). The calcium channels *trp *and *trpl *are normally considered to be eye-specific, and have an essential role in phototransduction [88-90]. It is thus not surprising to find both genes almost at the bottom of the gene list. We have shown, however, that fluid secretion is severely compromised by mutations in either gene. Similarly, nitric oxide synthase (NOS) is a major signal transducer in tubule [20,72]. Nonetheless, all three genes are within the 'bottom' 20 of the whole array, with signals that are barely detectable and significant depletion compared with the whole fly. This is a cautionary example: while abundant or enriched signals can be taken as reliable indicators of functional significance, the converse is not necessarily true.

### The tubule and human disease

**Figure 5:**
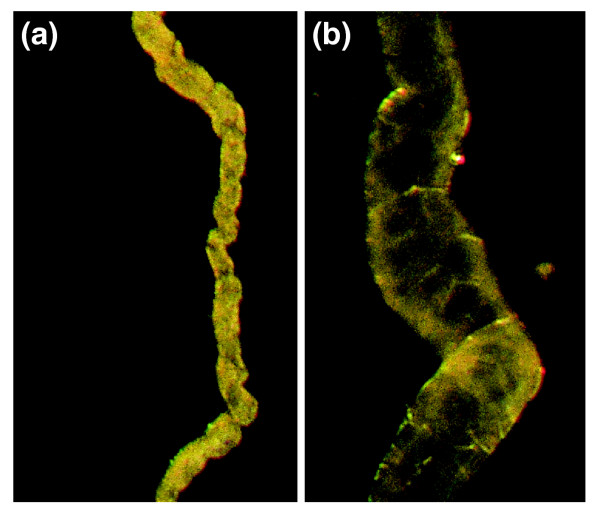
Recapitulation of human xanthinuria type 1 by *rosy *mutants. **(a) **Wild-type tubule; **(b) **tubule from adult *ry2 *homozygous fly. Both micrographs are at the same magnification, and the diameter of the wild-type tubule can be taken as 35 μm.

Consequent to the demonstration of the phenotype gap, there are some intriguing, abundant and enriched genes which by virtue of their non-uniform expression, are likely to be important in (and best studied in) tubule. A systematic approach was taken by combining the tubule-enriched gene list with the homophila database of *Drosophila *genes with known human disease homologs. The results (Table 14) show the 50 human diseases with *Drosophila *homologs that are upregulated at least threefold in tubules. Intriguingly, several of these genes have human kidney phenotypes. Some are extremely well studied: for example, *rosy *(one of the first *Drosophila *mutations recorded) encodes xanthine oxidase, and mutation in either human or fly produces severe nephrolithiasis with concomitant distortion of tubules (reviewed in [12]). The distension of tubules is remarkable (Figure 5). In both species, lethal effects can be ameliorated by a high-water, low-purine diet. Other diseases, although less well documented, have plausible renal phenotypes: for example, antenatal Bartter syndrome, a severe salt-wasting renal disease, associated with mutations in the ROMK channel (homolog *ir*); Dent disease, caused by mutation in *ClC5 *(homolog *CG5284*); proximal renal tubular acidosis, caused by mutation in the NDAE co-transport (homolog *ndae1*); nephrophatic cystinosis, caused by mutation in a lysosomal cystine transporter (homolog *CG17119*); mucopolysaccharidosis type IV, caused by mutation in galactosamine-6-sulphatase, an enzyme enriched in both human and fly kidney (homolog *CG7402*). Overall, there is a clear message that human and fly renal function may be relatively similar over quite a wide range of properties.

The tubule phenotype may also prove highly informative for other genes implicated in disease. Recently, a small 10 kDa protein, bc10, was shown to be downregulated in the transition from early-stage to invasive bladder carcinoma [91]. The normal function of this protein is not yet established, but its homolog (*bc10*) is highly abundant (893 ± 50) and moderately enriched (1.9 ± 0.09) in tubule, and a P-element insertion within the gene P{GT1}BG02443, is available from stock centers.

This comparative approach can be extended to non-human species. For example, *CG4928 *represents an abundant and enriched transcript (3,778, 13 times enriched), that is highly similar (1.9 × 10^-75^) to the *C. elegans *gene *unc-93 *[92]. This is associated with a 'rubber-band' phenotype, in which motor co-ordination is sluggish; it is thus taken to be a myogenic or neuromuscular gene. The discovery that a close homolog is highly enriched in renal tissue opens new lines of investigation for this gene.

## Discussion

**Figure 6:**
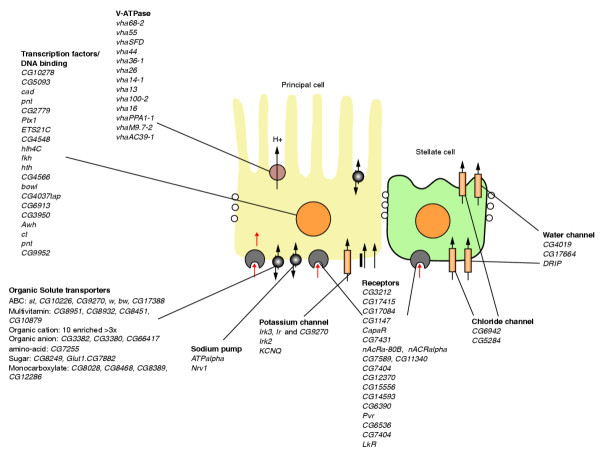
Summary of major genes enriched in tubule. Genes shown are upregulated at least threefold.

These data have value at two distinct levels: specific and general. Specifically, we have found out more about the operation of the Malpighian tubule than in any single published piece of work since the very first pioneering days: a summary is given in Figure 6. This tissue is of great interest, both for developmental studies and for integrative physiological study of epithelial function. Despite 990 papers on Malpighian tubules since the start of the twentieth century, and a really rather good understanding of ion and water transport, the microarray data provide strong indications that these are only minor properties of the tubule. Whole families of transporters are represented by abundant mRNAs and transport solutes that have yet to be studied in the context of tubule. Some datasets implicate particular genes in processes that have been studied in great physiological detail, and the presence of known genes with the novel can only increase our confidence in the result. In this context, the demonstrated abundance of transporters for almost every class of organic and inorganic solute dramatically diminishes the number of solutes for which a nonspecific paracellular pathway need be invoked. The data thus allow the conceptual view of the epithelium to alter from leaky to tight in a physiological-transport sense: this is consistent with electrophysiological data [93].

There are two areas where microarray data deserve comment. Firstly, more than 300 genes are expressed in tubule but called as absent in whole-fly samples. Although there is an obvious convenience and consistency in employing whole-organism samples for array studies, it is important to recognize that the approach is very likely to suppress the detection of those interesting genes that are not widely expressed. Secondly, the premise that abundance on an array (or more generally, abundance of an RNA species) necessarily correlates with functional significance can be spectacularly refuted by three examples, the *trp *and *trpl *channels and NOS. It is, however, probably significant that these are cell-signaling molecules, where a relatively small number of molecules can have a disproportionate influence on cell behavior. By contrast, the transport genes for which the tubule is so enriched are much more likely to exert effects proportional to their abundance.

## Conclusions

Reverse genetics is a vital tool in functional genomics, but the 'phenotype gap' has hampered widespread implementation of this approach [35]. As the tubule presents a range of easily assayed phenotypes [12], this work specifically identifies those genes that are likely to be best studied in tubule by virtue of their very high enrichment. In addition to the obvious transport genes, it is interesting that many transcription factors and human disease gene homologs fall into this category. This work thus stresses the importance of systematic, fine-grained, tissue-specific microarray analysis in closing the phenotype gap for multicellular model organisms.

## Materials and methods

### Flies

*Drosophila melanogaster *were kept on standard diet at 25°C and 55% relative humidity on a 12:12 h photoperiod. Malpighian tubules were dissected from 7-day-old adults, for compatibility with the extensive physiological literature on the tubule [10,11,13,15,17,19,20,39,70,75,94-96]. At this stage, the tubules are in a relatively stable state after adult emergence, and their secretion parameters do not change detectably between 3 and 14 days post-emergence.

### Microarrays

Tubules were dissected in batches of 1,000 by a group of eight experimenters. Tubules were aggregated into Trizol every 15 min to minimize the distortion of the transcriptome by the trauma of dissection and *in vitro *incubation. Care was taken to sever the tubules from the gut at the lower ureter so that no other tissue was included in the sample. For each experimental point, whole flies from the same culture were homogenized in Trizol in batches of 100, to permit a matched pair comparison. Six repeats were performed. RNA was extracted according to standard protocols, and quality was assessed with an Agilent RNA Bioanalyzer. Samples of 20 μg total RNA were reverse-transcribed, then *in vitro *transcribed, according to Affymetrix standard protocols. The quality of the ccomplementary RNA (cRNA) was also checked on an Agilent RNA Bioanalyzer, with a sample in which the broad cRNA peak exceeded the height of the low molecular weight degradation peak taken to be satisfactory. Samples were then run on the Affymetrix *Drosophila *genome array under standard conditions. Quality control was at several levels: the Affymetrix MAS 5.0 software provided evidence of successful sample preparation, with test genes providing a 3':5' signal ratio of less than 3. dChip [97] provided an alternative view, with a direct oligo-by-oligo view on the success of hybridization across the array surface; slides with both single-probe and probe-set outlier rates of less than 5% were taken as satisfactory. Only arrays in which both results were in range were accepted. In this case, 11 of 12 arrays were satisfactory; the first tubule array failed both MAS and dChip criteria, and so the first experimental pair was discarded to leave a five-sample paired design. As will be seen from the results, this design was sufficient to identify tubule-enriched genes with a high level of confidence. As sample collection extended over the whole day, array results from morning versus afternoon samples were compared (data not shown), but no difference was found between the two groups at this very broad time resolution.

### Bioinformatics

Microarray samples were analyzed by two independent routes. The first was low-level analysis with the Affymetrix MAS 5.0 suite and identification of differentially expressed genes using the Affymetrix Data Mining Tool. The second was low-level analysis using dChip software [97] followed by assessment of significance using SAM software [98] followed by post-analysis by dChip. The MAS5 low-level analysis consisted of background subtraction followed by robust conversion of probe-level perfect match-mismatch (PM-MM) expression values into probe-set-level signals followed by linear multi-chip normalization (scaling). Tubule enrichment was based on an Affymetrix 'up' call, and a critical level of *p *< 0.05. In this analysis method, tubule and fly samples were taken as matched pairs, reflecting their biological origin. The dChip-based low-level analysis consisted of background correction followed by the multi-chip, 'invariant-set' nonlinear normalization at probe level followed by the calculation of model-based expression indices using PM expression values only. Differentially expressed genes between two groups of five replicates were identified within dChip by filtering data using the following criteria: lower 90% confidence bound of fold-change [21] > 2; difference between group means on antilog scale > 100 and *p*-value for *t*-test of equal group means < 0.01. Alternatively, the differentially expressed genes were identified using SAM software with 1,000 sample permutations and false-discovery rate cutoff of 1%. These were then post-filtered using two first criteria from the dChip analysis mentioned above. Fold change was calculated as a ratio of group means. Outputs were saved as Excel files, and parsed by hand-coded Perl scripts.

## Additional data file

A list of genes (Additional data file 1) called as upregulated in tubule by Affymetrix SAM 5 software, and with more than two-fold enrichment is available with the online version of this article.

## Supplementary Material

Additional data file 1 A list of genes called as upregulated in tubule by Affymetrix SAM 5 software, and with more than two-fold enrichment Click here for additional data file

## Figures and Tables

**Table 1:** Most abundant genes in tubule, sorted by normalized Affymetrix signal strength

Gene	Signal	Enrichment	Function
*MtnA*	12,114 ± 581	3.0 ± 0.0	Cu-binding
*CG7874*	10,672 ± 518	7.4 ± 0.4	
*CG14292*	10,392 ± 572	8.4 ± 0.5	
*CG3168*	10,199 ± 459	6.2 ± 0.3	Transporter
*RpS25*	9,368 ± 276	1.3 ± 0.0	Small-subunit cytosol ribosomal protein
*Adh*	8,895 ± 395	1.3 ± 0.0	Alcohol dehydrogenase; EC 1.1.1.1
*RpS20*	8,720 ± 226	1.2 ± 0.0	Small-subunit cytosol ribosomal protein
*CG13315*	7,818 ± 370	3.9 ± 0.6	
*CG14245*	7,767 ± 305	13.4 ± 2.3	
*RpL27A*	7,757 ± 198	1.3 ± 0.0	Large-subunit cytosol ribosomal protein
*CG18282*	7,711 ± 160	1.7 ± 0.0	
*RpL18A*	7,514 ± 200	1.4 ± 0.0	Large-subunit cytosol ribosomal protein
*RpL14*	7,483 ± 209	1.3 ± 0.0	Large-subunit cytosol ribosomal protein
*RpP2*	7,481 ± 283	1.3 ± 0.1	Cytosolic ribosomal protein
*CG6726*	7,307 ± 244	14.4 ± 0.5	Peptidase
*RpL23a*	7,284 ± 254	1.2 ± 0.1	Large-subunit cytosol ribosomal protein
*CG4046*	7,250 ± 165	1.1 ± 0.1	Structural protein of ribosome
*CG7084*	7,211 ± 329	36.8 ± 6.5	Transporter
*RpL3*	7,179 ± 105	1.4 ± 0.1	Large-subunit cytosol ribosomal protein
*CG9914*	7,088 ± 466	12.0 ± 1.4	Enzyme
*CG3203*	7,024 ± 219	1.3 ± 0.1	L17-like
*CG6846*	6,989 ± 177	1.3 ± 0.1	Structural protein of ribosome
*blw*	6,890 ± 142	1.7 ± 0.0	ATP synthase alpha subunit
*BcDNA:GH08860*	6,742 ± 278	5.0 ± 0.3	Enzyme
*RpS3*	6,709 ± 240	1.3 ± 0.1	DNA-(apurinic or apyrimidinic site) lyase
*CG5827*	6,603 ± 169	1.3 ± 0.1	Structural protein of ribosome
*CG15697*	6,543 ± 174	1.3 ± 0.1	Structural protein of ribosome
*RpS9*	6,502 ± 171	1.2 ± 0.0	Small-subunit cytosol ribosomal protein
*Rack1*	6,463 ± 105	1.3 ± 0.0	Protein kinase C binding protein
*vha26*	6,416 ± 190	3.1 ± 0.3	V-ATPase E subunit
*Ser99Da*	6,305 ± 2100	0.6 ± 0.2	Serine carboxypeptidase
*Ser99Db*	6,300 ± 2119	0.6 ± 0.2	Serine-type endopeptidase
*CG1883*	6,258 ± 172	1.2 ± 0.1	Structural protein of ribosome
*RpL32*	6,251 ± 217	1.3 ± 0.1	Large-subunit cytosol ribosomal protein
*Atpalpha*	6,240 ± 151	4.2 ± 0.1	Na, K-ATPase alpha subunit
*CG3270*	6,234 ± 167	32.3 ± 2.6	Sarcosine oxidase
*RpS26*	6,080 ± 151	1.3 ± 0.1	Small-subunit cytosol ribosomal protein
*sop*	6,070 ± 157	1.1 ± 0.0	Small-subunit cytosol ribosomal protein
*RpL7*	6,060 ± 113	1.2 ± 0.0	Large-subunit cytosol ribosomal protein
*CG3321*	6,034 ± 122	1.6 ± 0.0	Enzyme
*eIF-4a*	6,027 ± 270	1.9 ± 0.1	
*CG8857*	5,977 ± 309	1.4 ± 0.1	Structural protein of ribosome
*oho23B*	5,940 ± 176	1.3 ± 0.1	Ribosomal protein
*CG3762*	5,874 ± 79	4.2 ± 0.1	
*CG9091*	5,850 ± 281	1.2 ± 0.1	Structural protein of ribosome
*vha16*	5,845 ± 215	2.6 ± 0.1	V-ATPase c subunit
*CG18323*	5,820 ± 201	1.5 ± 0.1	

**Table 2:** Genes enriched more than 25-fold in tubules

Gene	Product	MAS enrichment
*CG13365*		98.9
*CG14957*		95.9
*CG13905*		85.2
*CG13836*		80.6
*Irk3*	Potassium channel protein-like	80.3
*CG14963*		55.4
*CG3014*		54.0
*CG13161*		53.8
*CG17043*		49.9
*CG18095*		47.8
*CG13656*		45.5
*CG13311*		43.5
*CG17817*		40.9
*CG9434*		40.6
*CG17522*	Glutathione transferase	39.5
*CG15359*		38.7
*CG7084*	Organic cation transporter	36.8
*CG8028*	Monocarboxylate transporter-like	36.6
*CG8951*	Sodium-dependent multivitamin transporter-like	35.8
*CG3690*		34.8
*CG15406*	Sugar transporter	34.5
*CG14293*		33.5
*CG17028*		33.4
*CG3285*	Sugar transporter-like	33.0
*CG3270*		32.3
*scarlet*	ATP-binding cassette (ABC) transporter	32.3
*CG6529*	Sugar transporter-like	32.1
*CG2680*	4-nitrophenylphosphatase-like	31.2
*CG8620*		30.5
*CG15279*	Cation amino-acid symporter	30.1
*CG9509*		29.7
*CG14539*		29.3
*CG3382*	Organic anion transporter	29.3
*CG6602*		29.3
*CG5361*	Alkaline phosphatase-like	29.2
*CG8957*	Iodide symporter-like	29.1
*CG10006*		29.0
*CG15155*		28.9
*CG10226*	ATP-binding cassette transporter	28.3
*CG2196*	Sodium iodide symporter	27.7
*CG16762*		27.6
*CG14195*		27.4
*CG8125*	Aryldialkylphosphatase	27.4
*CG7881*	Sodium phosphate cotransporter	27.1
*CG8934*	Sodium iodide symporter-like	27.1
*CG7402*	*N*-acetylgalactosamine-4-sulfatase-like	26.9
*NaPi-T*	Na phosphate cotransporter	26.8
*CG8791*	Sodium phosphate cotransporter	26.8
*CG8776*	Cytochrome b561-like	26.6
*CG3212*		26.6
*CG14857*	Organic cation transporter-like	26.4
*CG8932*	Sodium-dependent multivitamin transporter-like	25.9
*Cyp6a18*	Cytochrome P450, CYP6A18	25.5

**Table 3:** Validation of array data by QRT-PCR

Gene	MAS enrichment	SAM enrichment	QRT-PCR enrichment
Highly enriched			
*CG13665*	98.9	8.7	9.0
*CG14957*	95.9	21.9	23.8
*CG13905*	22.6	17.4	110
*CG13836*	80.6	30.1	11.7
			
Evenly expressed			
CG17737	1.0	0.9	0.74
CG10731	1.0	1.1	0.68
CG8327	1.0	0.8	1.2
Arp66	1.0	1.1	0.47
			
Highly depleted			
*CG13421*	0.00	0.067	0.19
*CG12408*	0.01	0.11	0.14
*Act88F*	0.01	0.14	0.03
*CG15575*	0.01	0.082	0.008

Enrichment in tubule mRNA compared to whole fly mRNA, computed from the microarray dataset with MAS 5.0 or SAM (see text), were compared with real values obtained by QRT-PCR. Four separate fly and tubule samples were run with primers for each gene, and for *rp49*, a ribosomal gene generally considered to be invariant. RNA quantities were calculated, and the gene:*rp49 *ratio calculated for each sample pair. Tubule enrichment was calculated as the (gene:*rp49*)_tubule_/(gene:*rp49*)_fly_.

**Table 4:** Transporters sorted by class

Gene/class	Signal	Enrichment
ATP-binding cassette (ABC) transporter (6/46)		
*st*	1,521 ± 34	32 ± 2.8
*CG10226*	290 ± 25	28 ± 3.4
*CG9270*	422 ± 21	21 ± 2.7
*w*	798 ± 53	10 ± 1.4
*bw*	18 ± 2	4 ± 1.2
*CG17338*	72 ± 6	3 ± 0.2
		
Cationic amino-acid transporter (1/5)		
*CG7255*	308 ± 34	7 ± 0.8
		
Copper transporter (1/6)		
*CG7459*	374 ± 6	5 ± 0.6
		
Monocarboxylate transporter (4/14)		
*CG8028*	2,567 ± 82	37 ± 2.1
*CG8468*	1,377 ± 67	10 ± 0.7
*CG8389*	698 ± 38	4 ± 0.2
*CG12286 *(*kar*)	550 ± 15	3 ± 0.1
		
Multidrug efflux transporter (1/6)		
*CG8054 *(*now CG30344*)	1,366 ± 68	6 ± 0.4
		
Pyrimidine-sugar transporter of Golgi (1/1)		
*CG3874 *(*frc*)	877 ± 40	5 ± 0.3
		
Oligopeptide transporter (1/3)		
*CG9444*	517 ± 12	10 ± 1.2
		
Organic anion transporter (3/5)		
*CG3382*	1,076 ± 56	29 ± 3.3
*CG3380*	3,385 ± 126	24 ± 1.6
*CG6417*	678 ± 90	9 ± 2.4
		
Organic cation transporter (11/21)		
*CG7084*	7,211 ± 329	37 ± 6.5
*CG14857*	472 ± 13	26 ± 5.5
*CG17751*	1,331 ± 34	25 ± 4.2
*CG16727*	3,152 ± 200	23 ± 3.2
*CG17752*	4,847 ± 37	21 ± 2.1
*CG14856*	36 ± 5	7 ± 2.4
*CG3168*	10,199 ± 459	6 ± 0.3
*CG6231*	269 ± 30	5 ± 1.0
*CG7342*	20 ± 2	5 ± 1.5
*CG8654*	274 ± 29	4 ± 0.6
		
Reduced folate transporter (2/3)		
*CG14694*	584 ± 22	13 ± 1.6
*CG6574*	190 ± 8	4 ± 0.3
		
Sodium bicarbonate cotransporter (1/1)		
*CG4675 *(*Ndae1*)	531 ± 34	5 ± 0.5
		
Sodium-dependent inorganic phosphate cotransporter (1 / 20)		
*NaPi-T*	1,430 ± 428	27 ± 2.3
		
Sodium-dependent multivitamin transporter (4/5)		
*CG8951 *(now *CG31090*)	1,363 ± 30	36 ± 3.9
*CG8932*	2,106 ± 130	26 ± 1.4
*CG8451*	365 ± 10	4 ± 0.4
*CG10879 *(now *CG31668*)	6 ± 1	3 ± 0.7
		
Glucose transporter (3/17)		
*CG7882*	4,951 ± 171	16 ± 0.8
*CG8249*	302 ± 12	6 ± 1.0
*Glut1*	342 ± 24	3 ± 0.2
		
Sugar transporter (7/7)		
*CG15406*	5,322 ± 186	35 ± 2.8
*CG3285*	1,405 ± 55	33 ± 1.3
*CG6529 *(now *CG31272*)	3,774 ± 131	32 ± 4.8
*CG15407*	840 ± 44	25 ± 2.1
*CG14606*	1,210 ± 56	22 ± 2.0
*CG15408*	3,333 ± 194	21 ± 1.7
*CG8837*	1,277 ± 88	19 ± 2.9
		
Zinc transporter (4/6)		
*BG:DS07295.1 *(now *CG3994*)	3,608 ± 91	10 ± 1.0
*CG4334*	378 ± 19	5 ± 0.4
*CG17723*	919 ± 59	4 ± 0.3
*CG5130*	104 ± 10	4 ± 0.6

For brevity, only family members enriched by more than threefold are shown. For each grouping, the numbers in parentheses refer to the number of genes enriched in tubule, compared to the total number of such genes in the *Drosophila *genome, as classified by Gene Ontology. Where original gene names have been superseded by later annotations of the *Drosophila *genes, the new names are shown in parentheses.

**Table 5:** V-ATPase genes that are enriched in tubule

Subunit	Copy number	Genes	Affymetrix reference	Signal	Enrichment
V_1 _sector					
A	3	*vha68-1 *(*CG12403*)	142380_at	9 ± 2	0.5 ± 0.1
		*vha68-2 *(*CG3762*)	146305_at	**5,874 ± 79**	**4.2 ± 0.1**
		*vha68-3 *(*CG5075*)	146306_at	2 ± 0	0.04 ± 0.02
B	1	*vha55 *(*CG17369*)	153041_at	**2,304 ± 74**	**2.7 ± 0.1**
SFD (H)	1	*vhaSFD *(*CG17332*)	144191_at	**2,671 ± 66**	**4.4 ± 0.2**
C	1	*vha44 *(*CG8048*)	153422_at	**1,400 ± 74**	**3.5 ± 0.1**
D	3	*vha36-1 *(*CG8186*)	152480_at	**2,846 ± 154**	**4.5 ± 0.4**
		*vha36-2 *(*CG13167*)	147073_at	2 ± 0.4	0.1 ± 0.0
		*CG8310*	144407_at	29 ± 4	0.6 ± 0.09
E	1	*vha26 *(*CG1088*)	151930_at	**6,416 ± 190**	**3.1 ± 0.3**
F	2	*vha14-1 *(*CG8210*)	143625_at	**3,722 ± 105**	**3.2 ± 0.2**
		*vha14-2 *(*CG1076*)	149368_at	5.6 ± 1.6	1.5 ± 1.1
G	1	*vha13 *(*CG6213*)	144156_at	**2,952 ± 68**	**3.3 ± 0.1**
					
V_0 _sector					
A	5	*vha100-1 *(*CG1709*)	153997_at	155 ± 8	0.8 ± 0.0
		*vha100-2 *(*CG7679, CG18617*)	142661_at	**3,718 ± 157**	**5.4 ± 0.3**
		*vha100-3 *(*CG30329*)	not on array		
		*CG12602*	146249_at	306 ± 26	1.3 ± 0.1
		*vha100-4 *(*CG7678*)	141662_at	66 ± 3	0.24 ± 0.04
c	5	*vha16 *(*CG3161*)	141528_at	**5,845 ± 215**	**2.6 ± 0.1**
		*vha16-2 *(*CG32089*)*/vha16-3*	148578_at	32 ± 7	1.4 ± 0.22
		(*CG32090*)			
		*vha16-4 *(*CG9013*)	147341_at	18 ± 4	1.4 ± 0.6
		*vha16-5 *(*CG6737*)	146189_at	36 ± 7	0.6 ± 0.12
PPA1	2	*vhaPPA1-1 *(*CG7007*)	142158_at	**1,895 ± 79**	**4.1 ± 0.2**
(c")		*vhaPPA1-2 *(*CG7026*)	149926_at	57 ± 7	0.9 ± 0.1
M9.7	3	*vhaM9.7-1 *(*CG11589*)	154011_at	101 ± 9	**1.8 ± 0.0**
(e, H)		*CG1268*	148161_at	14 ± 1	0.1±0.0
		*vhaM9.7-2 *(*CG7625*)	149187_at	**3,101 ± 127**	**2.9 ± 0.1**
AC39	2	*vhaAC39-1 *(*CG2934*)	154279_at	2,082 ± 52	**3.4 ± 0.1**
(d)		*vhaAC39-2 *(*CG4624*)	150428_at	13 ± 2	0.8 ± 0.12

All genes significantly similar to known human or yeast V-ATPase subunits were identified by BLAST search, extending our previously reported annotation of the V-ATPase family [53], by identifying the genes underlined above as V-ATPase subunits. For comparison, enrichment ratios significantly greater than 1 and signals over 1,000 are shown in bold. (*vha16-2 *and *vha16-3 *are in tandem repeat and share the same Affymetrix oligo set, and so cannot be distinguished here.)

**Table 6:** Na^+^, K^+^-ATPase

Gene	Signal	Enrichment
α-subunit		
*Atpalpha*	6,240 ± 151	4.22 ± 0.05
*CG3701*	6 ± 1	0.85 ± 0.17
		
β-subunit		
*Nrv1*	1,924 ± 71	3.47 ± 0.21
*Nrv2*	2 ± 1	0.09 ± 0.06
*CG11703*	7 ± 2	0.46 ± 0.18
*CG5250*	4 ± 0	0.18 ± 0.04
*CG8663*	20 ± 1	0.1 ± 0.01

Although the *Drosophila *Na^+^, K^+^-ATPase has classically been thought to be composed of a dimer of *Atpalpha *and either *Nrv1 *or *Nrv2*, the other genes here are more similar by BLASTX to the corresponding alpha and beta subunits than any other gene (data not shown). They are thus included in the table as candidate alternative subunits.

**Table 7:** Potassium channels and symporters

Gene	Signal	Enrichment
Potassium channels		
*Irk3 (CG10369)*	2771 ± 145	80.31 ± 7.75
*Ir (CG6747)*	1302 ± 112	14.19 ± 1.58
*Irk2 (CG4370)*	527 ± 33	5.69 ± 0.24
*KCNQ (CG12215)*	101 ± 0	6.44 ± 2.31
*KCNQ (CG12915)*	111 ± 10	2.84 ± 0.46
*CG10864*	29 ± 7	3.74 ± 1.12
*CG32770 (CG6952)*	5 ± 2	2.6 ± 1.05
*elk*	5 ± 3	2.23 ± 1.19
*CG9361*	6 ± 2	2.31 ± 0.84
*CG12214*	101 ± 11	2.15 ± 0.51
*CG7640*	12 ± 5	1.48 ± 0.76
*eag*	8 ± 1	1.59 ± 0.39
*Shaker cognate b*	6 ± 1	1.38 ± 0.54
*CG4450*	4 ± 0	1.62 ± 0.28
*Shaw*	26 ± 4	1.21 ± 0.54
*CG1756*	15 ± 4	1.31 ± 0.35
*Shaker*	26 ± 4	1.42 ± 0.23
*CG9637*	3 ± 1	1.32 ± 0.22
*Shal*	29 ± 4	1.19 ± 0.29
*CG3367*	6 ± 1	1.09 ± 0.09
*CG8713*	41 ± 3	0.9 ± 0.1
*Sh*	7 ± 3	0.65 ± 0.25
*CG9194*	8 ± 1	0.77 ± 0.12
*CG15655*	13 ± 3	0.45 ± 0.13
*Ork1*	28 ± 2	0.32 ± 0.03
*sei*	10 ± 2	0.21 ± 0.04
*Shab*	6 ± 1	0.21 ± 0.04
*CG12904*	4 ± 1	0.14 ± 0.07
*CG17860*	5 ± 2	0.1 ± 0.04
*Hk*	4 ± 1	0.07 ± 0.01
		
Calcium-activated potassium channels		
*CG10706*	21 ± 5	1.93 ± 1.06
*slo*	2 ± 0	0.11 ± 0.02
*CG4179*	4 ± 1	1.55 ± 0.91
		
Potassium-dependent sodium-calcium exchangers		
*CG14744*	39 ± 1	1.32 ± 0.12
*CG1090*	35 ± 2	0.81 ± 0.13
*CG14743*	5 ± 1	0.48 ± 0.19
*Nckx30C*	31 ± 5	0.38 ± 0.05
*CG12376*	8 ± 2	0.24 ± 0.07
*Nckx30C*	31 ± 5	0.11 ± 0.05
		
Sodium/potassium/chloride symporter		
*EG:8D8.3*	132 ± 6	2.46 ± 0.36
*CG10413*	185 ± 25	1.75 ± 0.22
*CG5594*	65 ± 5	0.8 ± 0.07
*CG2509*	60 ± 6	0.42 ± 0.06
*CG4357*	12 ± 4	0.12 ± 0.04

**Table 8:** Chloride channels

Gene	Signal	Enrichment
*CG6942*	251 ± 9	4 ± 0.29
*CG8594*	57 ± 5	0.86 ± 0.09
*CG5284*	100 ± 5	2.2 ± 0.16

These are the three genes with clear similarity to the ClC gene family of vertebrates [12].

**Table 9:** Aquaporins and other major intrinsic proteins

Gene	Signal	Enrichment
*CG4019*	1666 ± 167	2.7 ± 0.3
*CG17664*	705 ± 91	7.9 ± 0.9
*DRIP*	318 ± 16	3.6 ± 0.4
*CG7777*	243 ± 11	0.6 ± 0.06
*CG12251 (AQP)*	22 ± 3	0.5 ± 0.04
*CG5398*	8 ± 1	0.2 ± 0.05
*bib*	2 ± 1	1.1 ± 0.3

**Table 10:** Receptors called as upregulated in tubule, with enrichments more than threefold

Gene	Signal	Enrichment
*CG3212*	85 ± 11	27 ± 11
*CG17415 *(calcitonin-like)	633 ± 48	17 ± 2
*CG17084*	288 ± 27	14 ± 2
*CG1147 *(neuropeptide Y-like)	34 ± 2	13 ± 8
*CG14575 *(CapaR)	311 ± 24	11 ± 1
*CG7431 *(octopamine-like)	40 ± 4	8.5 ± 0.9
*CG12414 (nAChRalpha @ 80B)*	9 ± 3	8 ± 3.6
*CG7589 *(ligand-gated Cl channel)	564 ± 35	7 ± 0.9
*CG12370 *(diuretic hormone-like)	203 ± 17	6.7 ± 9
*CG15556*	221 ± 12	6.4 ± 0.5
*CG11340 *(glycine-gated channel-like)	143 ± 8	5.0 ± 0.9
*CG14593 *(bombesin)	59 ± 13	5 ± 2
*CG6390 *(insulin-like growth factor)	85 ± 8	4.3 ± 0.6
*CG8222 *(*Pvr*, vascular endothelial growth factor-like)	294 ± 26	4.2 ± 0.5
*CG6536*	42 ± 5	4 ± 1.7
*nAcRalpha*	24 ± 4	4 ± 1.5
*CG7404 *(steroid-like)	239 ± 21	3.5 ± 0.4
*CG10626 *(LkR)	142 ± 7	2.9 ± 0.4

**Table 11:** Major genes of the cAMP, cGMP and calcium signaling pathways

Function	Gene name	Signal	Enrichment	Comments
cAMP				
Adenylate cyclase	*rutabage*	121 ± 12	1.4 ± .2	
	*Ac78C*	44 ± 5	7.2 ± 1.6	
	*Ac13E*	106 ± 4	4.1 ± 0.5	
Protein kinase A	*Pka-C3*	88 ± 9	1.7 ± 0.2	Catalytic subunit
	*Pka-R1*	183 ± 13	1.2 ± 0.1	Regulatory subunit
PDE	*dunce*	147 ± 6	3.9 ± 0.6	cAMP-specific
				
Calcium				
CamKinase	*Caki*	112 ± 10	1.7 ± 0.2	
Phospholipase C	*Small wing*	46 ± 6	1.1 ± 0.2	
	*Plc21C*	58 ± 5	1.1 ± 0.1	
Calcium release channels	*Itp-r83A*	11 ± 2	1.2 ± 0.2	InsP_3 _receptor
Calmodulin	*Calmodulin*	1,019 ± 57	0.9 ± 0.06	
Protein kinase C	*Pkc98E*	217 ± 15	1.7 ± 0.2	
				
cGMP				
Guanylate cyclase	*CG14885*	13 ± 3	6 ± 2.5	Probably soluble beta subunit
	*Gyc76C*	410 ± 23	2.9 ± 0.4	Membrane form
	*CG4224*	23 ± 4	0.8 ± 0.2	Membrane form
	*CG9873*	137 ± 5	2.0 ± 0.7	Membrane form
	*Gycbeta100B*	20 ± 4	0.8 ± 0.1	Cytoplasmic, beta subunit
	*CG5719*	9 ± 3	3.5 ± 1.4	Membrane form
PDE	*CG10231*	182 ± 4	3.7 ± 0.6	cGMP-specific, PDE11-like
Protein kinase G	*foraging*	91 ± 2	0.3 ± 0.01	
	*Pkg21D*	448 ± 20	15.7 ± 2.3	
				
Serine/threonine protein phosphatases				
	*Cg17746*	258 ± 32	4.3 ± 0.6	PPA-2C like
	*puckered*	228 ± 11	2.5 ± 0.2	Multifunctional
	*twins*	209 ± 11	2.0 ± 0.1	PPA-2A like
	*Pp2A-29B*	738 ± 28	1.9 ± 0.2	PPA-2A like
	*Microtubule star*	997 ± 46	1.3 ± 0.1	PPA-2A like
	*Pp1-87B*	318 ± 17	1.1 ± 0	PPA-1 like
	*Pp1alpha-96A*	332 ± 8	1.1 ± 0	PPA-1 like
				
Accessory proteins, associated with anchoring, cellular localization or modulation of signaling				
	*Akap550*	136 ± 6	2 ± 0.3	A-kinase anchor protein
	*AKAP200*	414 ± 30	0.35 ± 0.02	
	*14-3-3-zeta*	1,789 ± 42	2.6 ± 0.2	Diacylglycerol-activated PKC inhibitor
	*CG32812*	42 ± 4	2.7 ± 0.4	Calcineurin
	*Rack1*	6,463 ± 105	1.3 ± 0	Receptor for activated C-kinase

**Table 12:** Transcription factors and DNA-binding proteins that are abundant or enriched in tubule

Gene	Signal	Enrichment
*CG10278*	175 ± 7	24.1 ± 11.5
*CG5093*	50 ± 4	19.3 ± 6.2
*pnt*	63 ± 5	17.5 ± 4.8
*CG2779*	5771 ± 317	16.8 ± 0.7
*Ptx1*	183 ± 8	12.7 ± 2.2
*Ets21C*	51 ± 17	9.8 ± 3.1
*CG4548*	91 ± 4	8.8 ± 4.3
*HLH4C*	6 ± 1	7.7 ± 6.9
*fkh*	266 ± 26	7.2 ± 1.1
*hth*	162 ± 13	7.2 ± 0.7
*CG4566*	17 ± 2	7.1 ± 4.2
*bowl*	71 ± 5	7.1 ± 0.7
*CG4037*	5 ± 1	6.7 ± 2.4
*tap*	5 ± 1	6.0 ± 3.0
*CG6913*	5 ± 2	6.0 ± 5.5
*CG3950*	287 ± 21	5.4 ± 0.9
*Awh*	21 ± 4	4.8 ± 1.4
*CG1162*	8 ± 1	4.7 ± 2.1
*ct*	145 ± 12	4.6 ± 0.8
*CG14202*	10 ± 1	4.6 ± 1.5
*tsh (ae)*	65 ± 5	4.6 ± 0.8
*CG9952*	45 ± 11	4.5 ± 0.6
*sv*	16 ± 2	4.3 ± 1.8
*fd59A*	11 ± 3	4.3 ± 1.7
*CG11914*	31 ± 4	4.2 ± 1.7
*slp2*	4 ± 2	4.1 ± 3.1
*Lim3*	13 ± 3	4.0 ± 1.1
*CG6419*	18 ± 3	4.0 ± 0.4
*Tis11*	337 ± 17	3.9 ± 0.6
*nvy*	27 ± 4	3.9 ± 1.1

**Table 13:** Genes with known significance to tubule function, but very low abundance/enrichment scores

Gene name	Signal	Enrichment
*NOS*	1 ± 0	0.2 ± 0.04
*trpl*	9 ± 2	0.03 ± 0.01
*trp*	3 ± 1	0.02 ± 0.01

**Table 14:** *Drosophila *tubule as a model for human genetic disease

Gene	Affymetrix signal	Enrichment	Blast probability	OMIM reference	Human disease	Available fly stocks
*CG10226*	290 ± 25	28.3	1.00E-184	171050	Colchicine resistance	
*CG7402*	99 ± 4	26.9	2.00E-40	253000	Mucopolysaccharidosis IVA	
*Ir*	1,302 ± 112	14.2	1.00E-76	600359	Bartter syndrome, antenatal, 601678	
*ry*	655 ± 44	13.0	1.00E-184	607633	Xanthinuria, type I, 278300	
*Ptx1*	183 ± 8	12.7	6.00E-38	602669	Anterior segment mesenchymal dysgenesis and cataract, 107250	
*Fmo-1*	131 ± 11	12.0	9.00E-27	136132	[Fish-odor syndrome], 602079	
*CG4484*	504 ± 50	12.0	1.00E-49	606202	Oculocutaneous albinism, type IV, 606574	
*DS00004.14*	759 ± 54	10.6	1.00E-123	603470	Citrullinemia, 215700	
*CG9455*	355 ± 40	9.0	1.00E-42	107400	Emphysema; emphysema-cirrhosis, hemorrhagic diathesis due to	
*CG5582*	825 ± 49	8.5	1.00E-69	607042	Ceroid-lipofuscinosis, neuronal-3, juvenile, 204200	
*Cyp4d2*	1,008 ± 70	8.3	1.00E-27	107910	Gynecomastia, familial, due to increased aromatase activity	
*CG7433*	1,364 ± 50	7.4	1.00E-153	137150	GABA-transaminase deficiency	
*CG1140*	894 ± 26	7.3	1.00E-176	245050	Ketoacidosis due to SCOT deficiency	
*CG9547*	860 ± 34	7.0	1.00E-164	231670	Glutaricaciduria, type I	
*PhKgamma*	2,665 ± 152	6.9	1.00E-111	172471	Glycogenosis, hepatic, autosomal	
*CG4623*	382 ± 37	6.8	4.00E-28	606598	Charcot-Marie-Tooth disease, mixed axonal and demyelinating	l(3)j7B3
*CG12370*	203 ± 17	6.7	5.00E-40	138033		
*CG15556*	221 ± 12	6.4	6.00E-12	602851	Convulsions, familial febrile, 4, 604352	
*KCNQ*	101 ± 0	6.4	1.00E-108	602235	Epilepsy, benign, neonatal, type 1, 121200; myokymia with neonatal	
*CG17119*	852 ± 28	5.7	6.00E-74	606272	Cystinosis, atypical nephropathic; cystinosis, late-onset juvenile	
*CG7408*	168 ± 6	5.6	3.00E-27	300180	Chondrodysplasia punctata, X-linked recessive, 302950	
*Spat*	724 ± 39	5.1	2.00E-88	604285	Hyperoxaluria, primary, type 1, 259900	EP(x)1365
*CG8743*	1,001 ± 44	4.9	1.00E-100	605248	Mucolipidosis IV, 252650	
*CG14593*	59 ± 13	4.9	2.00E-33	131244	ABCD syndrome, 600501; Hirschsprung disease-2, 600155	
*CG1673*	911 ± 142	4.8	1.00E-100	113530	Hypervalinemia or hyperleucine-isoleucinemia (?)	
*Ndae1*	531 ± 34	4.7	1.00E-184	603345	Renal tubular acidosis, proximal, with ocular abnormalities, 604278	
*CG7834*	3441 ± 106	4.3	8.00E-80	130410	Glutaricaciduria, type IIB, 231680	EP(2)2553, l(2)k00405
*Pvr*	294 ± 26	4.2	6.00E-69	164770	Myeloid malignancy, predisposition to	
*CG12030*	887 ± 51	4.1	1.00E-124	606953	Galactose epimerase deficiency, 230350	
*Mdr49*	239 ± 25	4.0	1.00E-184	171060	Cholestasis, familial intrahepatic, of pregnancy, 147480	l(2)k05224
*CG4685*	563 ± 19	4.0	1.00E-129	271980	Succinic semialdehyde dehydrogenase deficiency	EP(2)2545, l(2)k08713
*CG12338*	774 ± 16	3.9	4.00E-40	124050		
*CG12582*	183 ± 14	3.8	1.00E-142	248510	Mannosidosis, beta-	l(2)k10108
*Reg-3*	463 ± 24	3.8	1.00E-184	274270	Thymine-uraciluria	
*Cyp12c1*	73 ± 5	3.8	2.00E-34	124080	Aldosterone to renin ratio raised; hypoaldosteronism, congenital,	
*Fur1*	724 ± 29	3.7	1.00E-163	162150	Obesity with impaired prohormone processing, 600955	
*Cyp9c1*	258 ± 14	3.7	7.00E-53	274180	Thromboxane synthase deficiency	l(3)05545
*Drip*	318 ± 16	3.6	1.00E-37	154050	Cataract, polymorphic and lamellar, 604219	
*CG8654*	274 ± 29	3.6	2.00E-62	607096	Hypouricemia, renal, 220150	
*Cyp9f2*	1,700 ± 60	3.6	1.00E-69	124010	CYP3A4 promoter polymorphism; CYP3A4-V	
*ERR*	239 ± 21	3.5	5.00E-29	313700	Androgen insensitivity, 300068; breast cancer, male	EP(3)3340
*CG3603*	94 ± 7	3.4	5.00E-20	222745	DECR deficiency (2) (?)	
*CG9232*	877 ± 20	3.4	1.00E-118	606999	Galactosemia, 230400	
*CG8417*	502 ± 31	3.2	3.00E-71	154550	Carbohydrate-deficient glycoprotein syndrome, type Ib, 602579	EP(2)0844, EP(2)2192, EP(2)2358, l(2)05428, l(2)k06503
*CG4663*	439 ± 14	3.2	2.00E-29	601789	Adrenoleukodystrophy, neonatal, 202370; Zellweger syndrome, 214100	
*Cat*	4,316 ± 88	3.2	1.00E-184	115500	Acatalasemia	
Prominin-like	308 ± 24	3.0	1.00E-20	604365	Retinal degeneration, autosomal recessive, prominin-related	EP(2)0740

Genes that are abundant (Affymetrix signal > 50) and enriched (> 3 times) in tubule, and which are also closely similar (Blast probablility < 10^-20^) to genes mutated in human genetic diseases, as described in the Homophila database [99]. OMIM reference refers to entries in the Online Mendelian Inheritance in Man database [100].
